# White Cord Syndrome After Cervical Laminoplasty in an 81-Year-Old Man

**DOI:** 10.7759/cureus.40386

**Published:** 2023-06-13

**Authors:** Satoshi Tanaka, Shinsuke Yoshida, Ryosuke Tomio, Akitake Mukasa, Terutaka Nishimatsu

**Affiliations:** 1 Neurosurgery, Numata Neurosurgery & Cardiovascular Hospital, Numata, JPN; 2 Neurosurgery, Saitama Medical Center, Kawagoe, JPN; 3 Neurosurgery, Honjo Neurosurgery and Spinal Surgery, Honjo, JPN; 4 Neurosurgery, Kumamoto University, Kumamoto, JPN

**Keywords:** motor evoked potential monitoring, t2-weighted mri, cervical decompression, cervical laminoplasty, white cord syndrome

## Abstract

White cord syndrome (WCS) shows high intramedullary signaling in T2-weighted MRI with worsening motor nerve symptoms after cervical spinal decompression surgery. It has been reported in only 13 cases.

An 81-year-old man had numbness, weakness, and impaired fine motor control in both upper limbs for the previous five years. C3, C4, C6, open-door laminoplasty, and C5 laminectomy were performed. Intraoperative transcranial motor evoked potential normalization by compound muscle action potential showed an 80% reduction in amplitude in the right abductor pollicis brevis and a 96% reduction in the right abductor hallucis. Tetraplegia occurred immediately after the operation. Magnetic resonance imaging (MRI) on the day after the operation showed intramedullary T2 high signals at the C4 and C5 levels. According to Brunnstrom's staging, the upper and lower right limbs and the lower left limb were at stage two, and the upper left limb was at stage three, six months after the operation. Thirteen cases of WCS have been reported in the literature. These were thought to be caused by reperfusion due to decompression.

## Introduction

White code syndrome (WCS) is a very rare postoperative cervical complication, with only 13 reported cases at the time of writing. [1-13]. It is believed to be the result of reperfusion injury after spinal decompression surgery in the absence of intraoperative damage, causing neurological deterioration. [13] The presence of intramedullary hyperintense regions without other pathological signs on postoperative T2-weighted magnetic resonance imaging (MRI) is characteristic of WCS. We report here the additional case that was thought to be WCS.

## Case presentation

**Figure 1:**
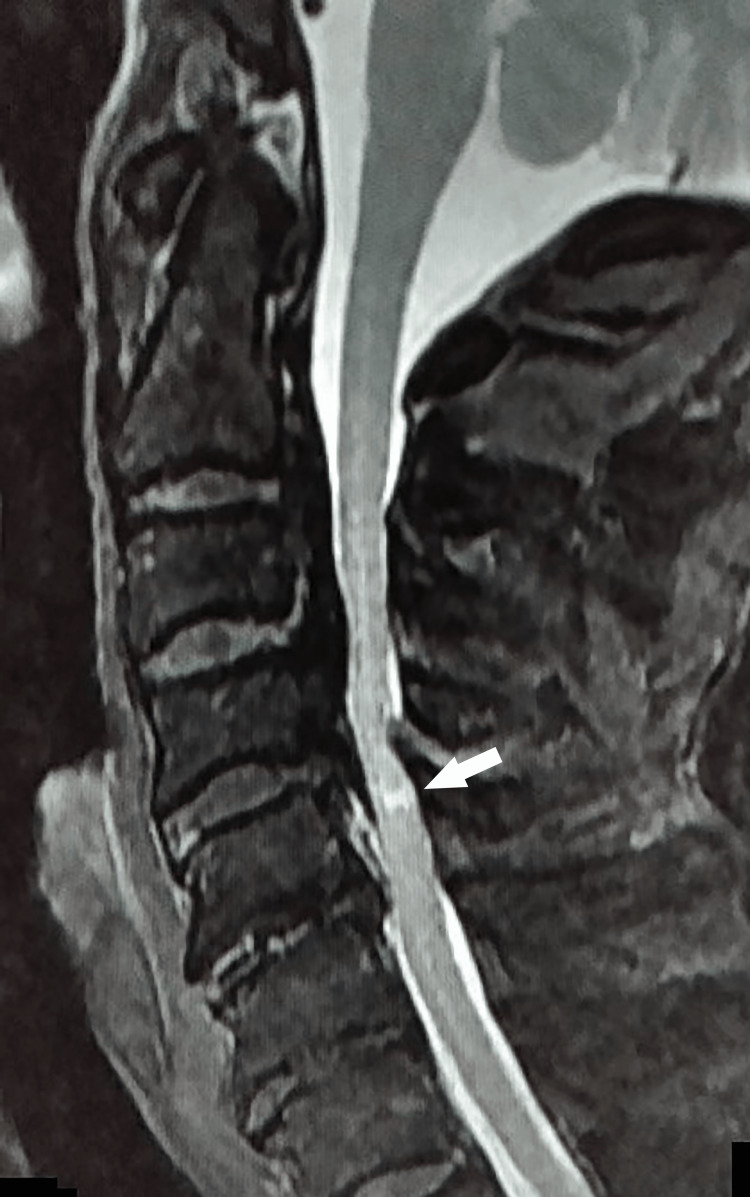
Preoperative T2-weighted cervical spine sagittal MRI Preoperative MRI revealed a severe spinal canal stenosis due to an ossified posterior longitudinal ligament at C3–C5, accompanied by intramedullary high signals in the T2-weight image (arrow).

An 81-year-old man reported having had numbness, weakness, and impaired fine motor control in both upper limbs for about five years. His cervical spine MRI revealed severe spinal canal stenosis due to an ossified posterior longitudinal ligament (OPLL) of C3-C5, accompanied by an intramedullary high signal in T2-weighted images (Figure 1). His preoperative Japan Orthopedic Association (JOA) score was 11 points.

**Figure 2:**
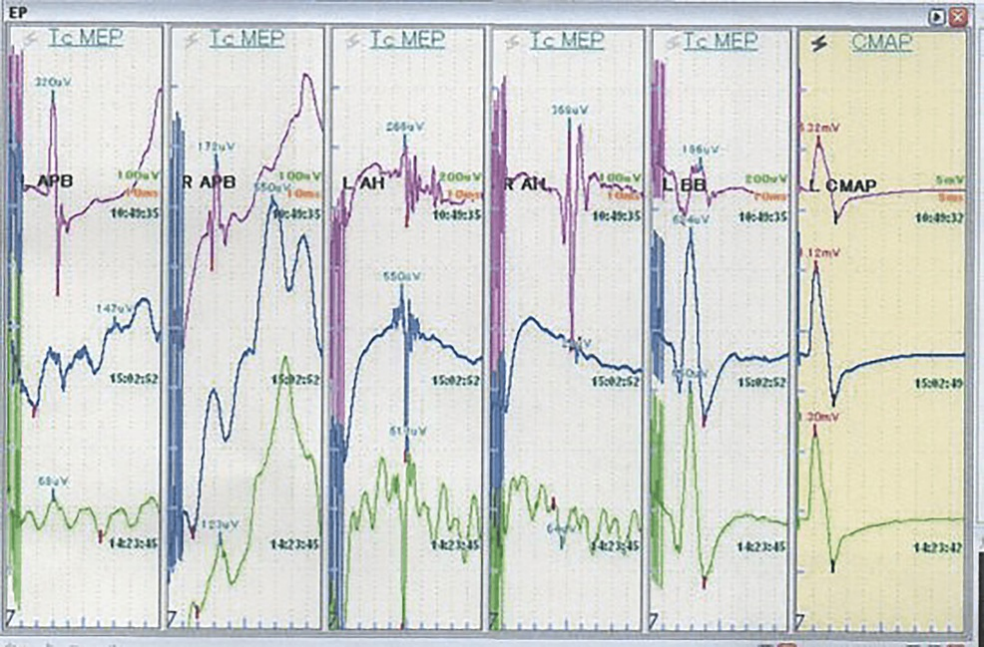
Intraoperative motor evoked potential monitoring Intraoperative motor evoked potential monitoring showed a marked reduction in amplitude of more than 70% (arrows) in bilateral abductor pollicis brevis muscles and of more than 95% (arrowhead) in bilateral abductor hallucis muscles under compound muscle action potential after peripheral nerve stimulation normalization.

C3-C6 double-door laminoplasty using the Laminoplasty Basket (Amtec Co., Ltd., Tokyo, Japan) was performed. As a result, a C5 laminectomy was performed due to a fracture of the C5 laminae. Intraoperative motor evoked potential (MEP) monitoring showed a marked reduction in amplitude of more than 70% in the bilateral abductors pollicis brevis and of more than 95% in the bilateral abductor hallucis under compound muscle action potential (CMAP) after peripheral nerve stimulation normalization (Figure 2) [14].

**Figure 3:**
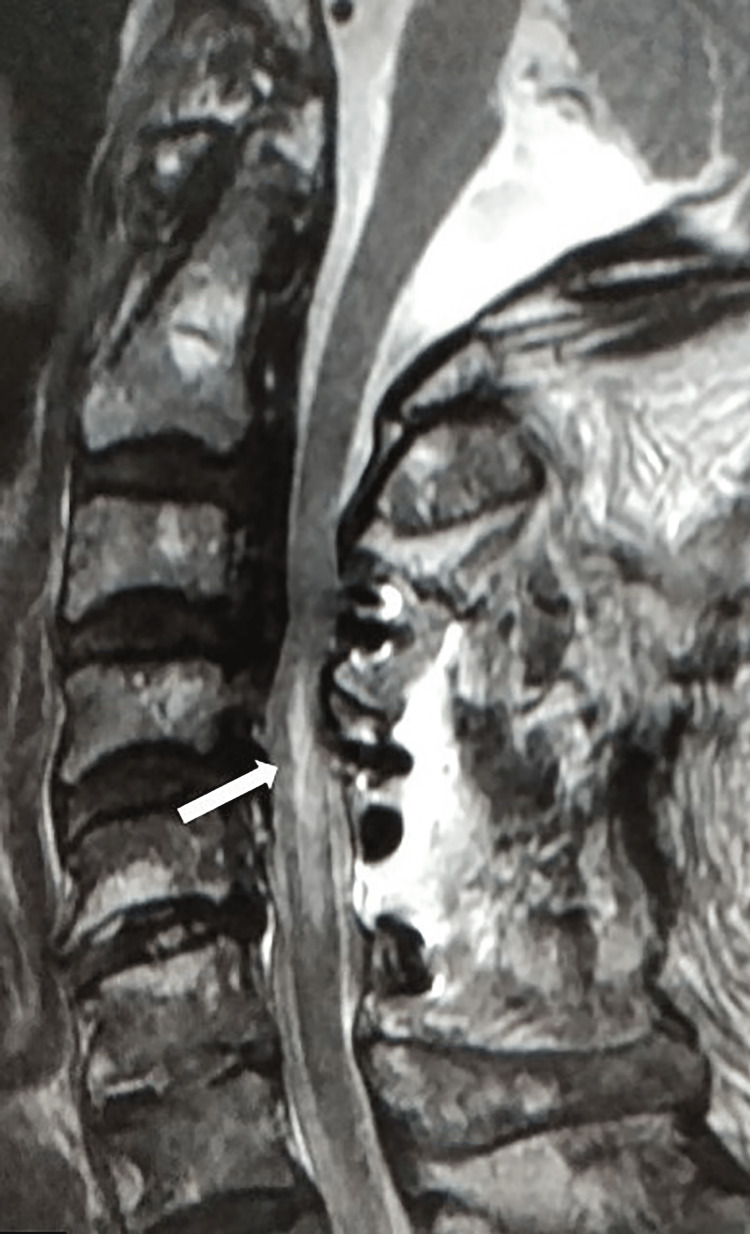
Postoperative sagittal T2-weighted MRI Postoperative MRI disclosed a marked intramedullary T2-high signal intensity at the C4-C5 level (arrow).

Strong quadriplegia was recognized, especially on the right side, which was consistent with the findings of MEP monitoring immediately after the operation. The decompression on CT was sufficient, and the follow-up consisted of only steroid administration. An MRI performed the day after the surgery revealed marked intramedullary T2-high signals at the C4 and C5 levels (Figure 3).

The postoperative JOA score was -0.5 points. He was transferred to a recovery-phase rehabilitation hospital and discharged to his home six months later, but the Brunnstrom stage was two for the upper right and lower left limbs and three for the upper left limb.

## Discussion

WCS was first reported in 2013; six cases were reported in 2020, and a total of only 13 cases have been described overall (Table 1) [1-13], including two cases of anterior cervical decompression with fusion and 11 cases of posterior decompression and/or fusion.

**Table 1:** Demographics and clinical characteristics of other cases of white cord syndrome reported ACDF: anterior cervical discectomy and fusion

No.	Year	Author	Approach	Palsy	Reoperation	Outcome
1	2013	Chin KR et al. [1]	ACDF(C4/5, C5/6)	Tetraparesis	ー	Poor
2	2018	Antwi P et al. [2]	Posterior	Left hemiparesis	ー	Moderate
3	2018	Vinodh VP et al. [3]	Posterior	Tetraplegia	ー	Poor
4	2019	Papaioannou I et al. [4]	Posterior	Paraparesis	＋	Moderate
5	2019	Wiginton JG et al. [5]	Posterior	Tetraplegia	ー	Good
6	2020	Jun DS et al. [6]	ACDF(C6/C7)	Paraplegia	＋	Good
7	2020	Mathkour M et al. [7]	Posterior	Right hemiparesis	ー	Moderate
8	2020	Sepulveda F et al. [8]	Posterior	Right arm monoplegia	ー	Good
9	2020	Liao YX et al. [9]	Posterior	Left hemiplegia	ー	Good
10	2020	Busack CD et al. [10]	Posterior	Tetraplegia	ー	Good
11	2020	Kalidindi KKV et al. [11]	Posterior	Tetraplegia	ー	Poor
12	2021	Segal DN et al. [12]	Posterior	Acute tetraplegia	ー	Good
13	2021	Malinovic M et al. [13]	Posterior	Tetraparesis	ー	Poor

There were seven cases of quadriplegia, two paraplegias, three hemiplegias, and one monoparalysis. Only two patients underwent emergency reoperation, and the other patients were treated with high-dose steroids. In particular, regarding post-decompression, in most cases sufficient decompression had already been performed, and it is considered that few cases needed further dural plasty or myelotomy. Neurological symptoms completely recovered in six patients, partially recovered in three patients, and never recovered in four patients. Nearly half of the cases of WCS were irreversible and had a poor prognosis.

In a survey of the prognosis of laminoplasty in 581 cases of OPLL by the Japanese Orthopedic Association published in 2011, before WCS was first reported, the neurological symptoms in seven patients (1.2%) became worse after six months [15]. Especially in posterior decompression of the cervical spine, it is generally considered that 1%-2% of cases develop a poor prognosis. Given the sudden increase in WCS reports over the past two to three years, it is likely that far more cases of WCS have been experienced by spinal surgeons than have been reported so far.

WCS is thought to cause edema and bleeding due to reperfusion syndrome as a result of rapid decompression [10,16]. Hyper-perfusion, hyperemia, and edema of the spinal cord are considered to occur when compression of a particularly strong spinal cord is rapidly lifted. This etiology and the disease state can be avoided because steroids are effective in many cases. [5,6,8-10,12]

A newly developed neurological deficit that may be attributed to an intraoperative iatrogenic cord injury is not WCS. Acharya et al. reported a case of quadriplegia following cervical posterior decompression surgery that had been caused by an intraoperative iatrogenic spinal cord injury, and WCS was misdiagnosed [17]. WCS is defined as an obvious postoperative motor neuropathy with an unexpected cause. Postoperative intraoperative iatrogenic surgeon-based mechanical cord injury, implantation extrusion, and failure to adequately resect OPLL, thus stretching the cord over residual disease, must first be ruled out. Of the 11 reported cases of WCS, two cases in which some improvement was observed by reducing residual OPLL pressure in a reoperation should not have been WCS [4,7]. If WCS is due to reperfusion caused by decompression, it is unlikely that further decompression surgery will improve the symptoms. Our presented case cannot deny the possibility of excessive decompression due to a fracture or freeing of the fifth cervical vertebral arch during surgery; this is often experienced in laminoplasty and cannot be determined to be the cause of the rare white cord syndrome. It is not clear how much a surgical procedure causes iatrogenic postoperative paralysis.

MEP monitoring under CMAP normalization after peripheral nerve stimulation had a sensitivity of 100% in spinal surgery and seemed to be very useful for predicting postoperative motor paralysis such as WCS during an operation [14,16]. However, our CMAP-normalized MEP monitoring is not 100% specific, and there is a possibility of false positives even if the amplitude decreases during surgery [18]. Furthermore, it is difficult to judge whether treatment such as high-dose steroid administration or dural plasty will be added.

In the case described here, the preoperative JOA score was more than 10 points. The risk of developing WCS may be higher in cases where the neurological symptoms are mild, even if the spinal cord is under strong compression in the imaging. The operation indication must be carefully judged, and sudden decompression in the operation must be avoided, especially during a posterior approach in cases where the neurological symptoms are mild even if the compression on the image is strong, as in the present cases.

## Conclusions

WCS has been reported in 14 cases, including our case. WCS is caused by reperfusion, and it seems to be important to avoid rapid decompression in cases with advanced cervical cord compression.
